# Liver fibrosis according to diabetes status and relation to cardiovascular risk and mortality in US adults

**DOI:** 10.1016/j.ahjo.2024.100457

**Published:** 2024-09-06

**Authors:** Matthew Bang, Wenjun Fan, Nathan D. Wong

**Affiliations:** Heart Disease Prevention Program, Division of Cardiology, University of California, Irvine, USA

**Keywords:** Diabetes, Liver fibrosis, Cardiovascular disease, Epidemiology

## Abstract

**Study objective:**

Liver fibrosis is associated with increased cardiovascular disease (CVD) risk and mortality. However, it is unknown how these risks compare in those with pre-diabetes (pre-DM) or diabetes (DM). We examined the association of FIB-4 levels, an indicator of liver fibrosis, with CVD risk and mortality according to DM status.

**Design and setting:**

Prospective, longitudinal cohort study.

**Participants:**

We examined 13,326 U.S. adults (6.7 % with DM) with FIB-4 measures classified as low (<1.30), intermediate (1.30- < 2.67), high (2.67- < 3.25), and very high (≥3.25). National Death Index linkage provided mortality status for CVD, liver-related, and all causes over 17.5 years.

**Main outcomes:**

We calculated 10-year ASCVD risk in persons without known ASCVD. Cox regression examined the relation of FIB-4 with mortality by DM status.

**Results:**

High/very high FIB-4 levels were greater in those with (2.2 %) vs. without (0.4 %) DM (*p* < 0.0001). Higher FIB-4 scores and DM were associated with greater estimated ASCVD risks (*p* < 0.0001); 44.5 % of those at high /very high FIB-4 levels had ≥20 % estimated ASCVD risk. CVD mortality hazard ratios (HRs) (95 % CI) associated with high/very high FIB-4 in those with pre-DM and DM were 8.76 (3.66–20.95), and 0.89 (0.22–3.53), respectively, and for total mortality were 5.46 (3.16–9.43), and 2.07 (0.90–4.74), respectively, which were attenuated after adjustment.

**Conclusions:**

Our findings indicate the need for increased efforts to identify those at risk of liver fibrosis in adults with pre-DM or DM to prevent CVD and total mortality.

## 1 Introduction

Nonalcoholic fatty liver disease (NAFLD) comprises a spectrum of conditions ranging from nonalcoholic fatty liver (NAFL) to nonalcoholic steatohepatitis (NASH), fibrosis, and eventually cirrhosis [1]. In the U.S. adult population, the prevalence of NAFLD (based on mild to severe steatosis by ultrasonography) has been estimated at 34 %, projecting to approximately 43.2 million adults [2]. With these numbers continuing to rise, there is growing concern for complications of NAFLD. Particularly concerning is the high risk of developing end-stage liver disease in persons with liver fibrosis [3]. The progression of NAFLD can irreversibly lead to liver fibrosis, and NAFLD has consistently been associated with a high risk of both diabetes and cardiovascular disease (CVD) [4].

Non-invasive measures of liver fibrosis include the NAFLD fibrosis score (NFS) and FIB-4 score which are used to identify advanced fibrosis. While NAFLD in general does not consistently predict total or cardiovascular mortality in multivariable analyses, both the NFS and FIB-4 scores strongly predict total and cardiovascular mortality in the general population [2]. Others have previously shown NAFLD with, but not without advanced fibrosis to predict total mortality [5] and NAFLD to relate to total and liver mortality [6]. Furthermore, there have been substantial increases in the prevalence of NASH cirrhosis from 1999-2002 to 2009-2012 (0.072 % vs. 0.178 %, respectively) as well as NAFLD with advanced fibrosis (0.84 % vs. 1.75 %, respectively); there were also significant increases in obesity, diabetes, and insulin resistance during this time [7]. Others have also shown fibrosis to be more common in those with NAFLD, with increasing age, obesity, and concurrent diabetes associated with an increased risk of fibrosis [8]. Moreover, diabetes has been shown to be associated with a near doubling in the risk of advanced fibrosis based on a FIB-4 score ≥ 2.67 (7.1 % vs. 3.8 %) [9]. In U.S. adults, the prevalence of pre-diabetes (pre-DM) and diabetes (DM) has increased over recent years to an estimated 91.8 million and 35.4 million adults, respectively, and these conditions together are present in approximately half of the U.S. adult population [10].

While liver fibrosis is associated with increased cardiovascular disease (CVD) risk overall, the extent to which this relation may be enhanced in those with pre-DM or DM has not been examined. The objectives of this study were to 1) examine how the extent of liver fibrosis varies according to the presence of pre-DM and DM, 2) estimate the atherosclerotic CVD risk according to the extent of liver fibrosis in those with and without Pre-DM and DM, and 3) examine CVD and total mortality according to the extent of liver fibrosis in those with and without pre-DM and DM.

## 2 Materials and methods

### 2.1 Study sample

We used data from the U.S. National Health and Nutrition Examination Surveys 1988–1994 (NHANES III), the advantage being the long follow-up time through 2015 for mortality outcomes. The methodology for data collection in NHANES surveys has been described extensively [11]. Persons included in this study were adults aged 20 and over with triglyceride (TG) data for morning fasting sessions (at least 8.5 h) and without known CVD or history of viral hepatitis B or C. Known CVD was determined by self-reported history of heart attack, stroke, heart failure, or coronary heart disease. History of viral hepatitis B was determined by analyzing laboratory data for positive hepatitis B surface antigen, and history of viral hepatitis C was determined by positive hepatitis C virus (HCV) RNA results. We excluded those with missing mortality data, missing follow-up time, excessive alcohol consumption, and lack of available FIB-4 data according to platelet count, aspartate aminotransferase (AST) measurements, and alanine aminotransferase (ALT) measurements. After exclusions, we studied in NHANES III 13,326 adults aged 20 and over projecting to 146.7 million adults in the U.S. population based on NHANES six-year sample weighting, with 1406 adults (6.7 %) having DM (projected to 9.8 million adults). The current project utilized de-identified publicly available data from NHANES which does not qualify for human subject research and is exempt from IRB review.

### 2.2 Measures

We calculated FIB-4 scores using the following formula: FIB-4 = [age (years) x AST (IU/L)]/ platelet count (10^9^/L) x [ALT (IU/L)]^1/2^ [2]. Previously published cutpoints were used to divide the sample into four FIB-4 categories: low (<1.30), intermediate (1.30- < 2.67), high (2.67- < 3.25), and very high (≥3.25) probability of advanced liver fibrosis [3]. Pre-diabetes was characterized by a fasting glucose measurement of 100–125 mg/dL or HbA1c measures of 5.7- < 6.5 %. Adults were defined as having diabetes after meeting at least one of the following criteria: (1) fasting glucose ≥126 mg/dL; (2) non-fasting glucose ≥200 mg/dL; (3) HbA1c ≥6.5 %; (4) taking insulin; (5) taking medication to lower blood sugar; or (6) self-reported DM. Weight categories, hypertension, metabolic syndrome, non-alcoholic fatty liver disease (NALFD) and excessive alcohol consumption [12] are defined as noted in Table 1.

**Table 1:** Definition of formulas and categories for FIB-4 measures and associated risk factors.

Name	Formula
FIB-4 score calculation	FIB-4 = [age (years) x AST (IU/L)] / platelet count (10^9^/L) x [ALT (IU/L)]^1/2^
FIB-4 categories	low (<1.30) intermediate (1.30- < 2.67)
	high (2.67- < 3.25) very high (≥3.25)
Diabetes Status	Pre-diabetes: Meets one or more of the following criteria: 1. Fasting glucose = 100–125 mg/dL 2. HbA1c 5.7- < 6.5 %
	Diabetes: Meets at least one of the following criteria: 1. Fasting glucose ≥126 mg/dL 2. Non-fasting glucose ≥200 mg/dL 3. HbA1c >6.5 % 4. Taking insulin 5. Taking medication to lower blood sugar 6. Self-reported DM
Weight	Normal weight: BMI < 25 kg/m^3^
	Overweight: 25 ≤ BMI < 30 kg/m^3^
	Obese: BMI ≥ 30 kg/m^3^
Hypertension	Meets one or more of the following criteria: 1. Blood pressure ≥ 130/80 mmHg 2. Taking medication for high blood pressure
Metabolic syndrome	Meets three or more of the following criteria: 1. Waist circumference > 88 cm for females or > 102 cm for males 2. Triglyceride levels ≥150 mg/dL 3. HDL-C < 50 mg/dL for females and < 40 mg/dL for males 4. Fasting glucose ≥100 mg/dL, taking insulin, or taking other diabetes medication 5. Blood pressure ≥ 130/85 mmHg or on antihypertensive medication Based on Alberti KG, et al. Circulation 2009;120(16):1640–5. PMID: 19805654.
Non-alcoholic fatty liver disease	Meets the following criteria: 1. ALT levels >20 U/L in women or >30 U/L in men 2. Has metabolic syndrome (with the exclusion of hepatitis B, hepatitis C, or alcoholism)
Excessive alcohol consumption	Meets at least one of the following criteria: 1. Underage drinking: any alcohol use under the age of 21 2. Heavy drinking: 15 or more drinks^a^ per week for men and 8 or more drinks per week for women 3. Binge drinking: 5 or more drinks on one occasion for men and 4 or more drinks on one occasion for women 4. Pregnant drinking: any alcohol consumed by pregnant women

a A drink is defined in NHANES III as a glass or can of beer, glass of wine, shot of hard liquor or mixed drink. https://wwwn.cdc.gov/nchs/data/nhanes3/manuals/mecint.pdf.

### 2.3 Mortality assessment and follow-up

The National Center for Health Statistics (NCHS) has linked data collected from several NCHS population surveys with death certificate records from the National Death Index (NDI) to provide U.S. mortality data including causes of death. We utilized NCHS mortality files for NHANES III 1988–1994 containing information on mortality from diseases of the heart, cerebrovascular system, liver-related, and all causes through December 2015.

### 2.4 Statistical analyses

We examined the number and proportion of U.S. adults within each FIB-4 group according to the presence of pre-DM and DM. We then examined demographic factors including age, sex, and ethnicity along with clinical and risk factor measures such as BMI, waist circumference, LDL-C, HDL-C, triglycerides, systolic and diastolic blood pressure, cigarette smoking, and alcohol use. The Chi-square test of proportions, ANOVA, Fisher's exact test, and generalized linear models were used to assess possible significant differences in these variables between the FIB-4 groups, as appropriate. Multiple logistic regression analyses were performed in those with and without DM as well as in the overall sample to determine predictors of being at high or very high FIB-4. In these logistic regression models, we adjusted for age, sex, ethnicity, total cholesterol, systolic and diastolic blood pressure, smoking status, alcohol use, the presence of NAFLD, and obesity.

The American College of Cardiology/American Heart Association Pooled Cohort atherosclerotic CVD (ASCVD) Risk Calculator [13] was used to determine the 10-year risk (%) of hard ASCVD (including myocardial infarction, stroke, and cardiovascular mortality). We then calculated the mean 10-year ASCVD risk according to FIB-4 category in those aged 40–75 without known ASCVD. Mortality rates for cardiovascular and all-causes were calculated per 1000 person years according to FIB-4 category in those with and without pre-DM or DM. The incidence of CVD and total mortality (per 1000 person years) from the National Death Index linkage of NHANES with follow-up through December 2015 was determined across FIB-4 categories in those with and without pre-DM and DM. Cox proportional hazards regression examined the risk of CVD and total mortality across FIB-4 category (with <1.30 as the reference) in those with and without pre-DM or DM with hazard ratios and 95 % confidence limits were calculated for both unadjusted and adjusted models including covariates not part of the FIB-4 definition: sex, ethnicity, BMI, non-HDL-C, HDL-C, hypertension, cigarette smoking, and alcohol use.

All analyses were done using SAS version 9.4 with the appropriate weighting factor to project the study sample to the U.S. population. By using NHANES data, we were able to utilize sample weights for each patient that are representative of the U.S. civilian noninstitutionalized resident population.

## 3 Results

We included 13,326 adults (projected to 146.7 million) from the NHANES 1988–1994 surveys aged 20 years and over who met the entry criteria and did not have missing data or non-positive sample weights (which indicate the number of individuals in the population represented). Overall 1406 (projected to 9.8 million) were defined to have diabetes, 3544 (31.2 M) pre-diabetes, and 8376 (105.6 M) had neither condition. Table 2 showed the distribution of demographic and clinical factors across FIB-4 categories. Overall, 84.4 % (projected to 130.7 million) had low levels of FIB-4 (<1.30) with 0.5 % (projected to 0.6 million) having high and 0.4 % (0.2 million) having very high FIB-4 (≥3.25). Those with high or very high FIB-4 levels comprised 0.5 % and 0.2 %, respectively, of those with MetS (*p* < 0.0001), 1.7 % and 0.5 % of those with DM, 1.0 % and 0.1 %, respectively, of those with NAFLD, and 0.3 % and 0.1 % of those with obesity. Those with higher FIB-4 scores were older in age and had greater waist circumference (*p* < 0.0001).

**Table 2:** Demographic characteristics of participants across FIB-4 categories.

	Level of FIB-4				*p*-value
	Low (FIB-4 < 1.30) (*n* = 11,249; 130.7 M, 89.2 %)	Intermediate (1.30 ≤FIB-4 < 2.67) (n = 1961; 15.1 M, 10.3 %)	High (2.67 ≤FIB-4 < 3.25) (*n* = 67; 0.6 M, 0.4 %)	Very high (FIB-4 ≥3.25) (*n* = 49; 0.2 M, 0.1 %)	
**Age (yr)**	40.7 ± 0.3	67.7 ± 0.6	66.9 ± 2.8	66.0 ± 2.5	<0.0001
**Sex**					
Male	4906 (60.6 M, 88.0 %)	1080 (8.0 M, 11.6 %)	33 (0.2 M, 0.3 %)	23 (0.1 M, 0.1 %)	<0.0001
Female	6343 (70.1 M, 90.2 %)	881 (7.1 M, 9.1 %)	34 (0.4 M, 0.5 %)	26 (0.1 M, 0.2 %)	
**Ethnicity**					
Non-Hispanic White	4327 (100.3 M, 88.2 %)	1216 (12.8 M, 11.2 %)	50 (0.5 M, 0.5 %)	20 (0.2 M, 0.1 %)	<0.0001
Non-Hispanic Black	3213 (13.6 M, 92.6 %)	312 (1.0 M, 6.9 %)	9 (0.03 M, 0.2 %)	11 (0.03 M, 0.2 %)	
Mexican American	3256 (6.9 M, 93.2 %)	373 (0.5 M, 6.5 %)	7 (0.004 M, 0.1 %)	16 (0.02 M, 0.2 %)	
Other Race	453 (9.8 M, 91.6 %)	60 (0.9 M, 8.2 %)	1 (0.003 M, 0.03 %)	2 (0.03 M, 0.2 %)	
**BMI (kg/m**^**2**^**)**	26.4 ± 0.1	27.1 ± 0.2	26.1 ± 0.9	25.1 ± 1.3	0.0051
**Waist circumference (cm)**	90.9 ± 0.2	97.4 ± 0.5	95.0 ± 2.2	90.7 ± 3.6	<0.0001
**LDL-C (mg/dL)**	126.6 ± 0.9	133.2 ± 2.0	122.0 ± 8.8	125.5 ± 17.0	0.0244
**HDL-C (mg/dL)**	50.8 ± 0.4	51.5 ± 0.7	59.5 ± 6.0	58.3 ± 4.4	0.0877
**Triglycerides (mg/dL)**	137.6 ± 2.4	166.1 ± 5.6	194.4 ± 46.7	124.3 ± 13.4	<0.0001
**SBP (mm Hg)**	119.7 ± 0.3	137.2 ± 0.8	134.0 ± 2.6	133.1 ± 2.9	<0.0001
**DBP (mm Hg)**	74.0 ± 0.2	75.5 ± 0.3	72.3 ± 1.1	74.4 ± 1.5	0.0018
**Smoking status**					
Yes	3023 (38.2 M, 94.8 %)	241 (2.0 M, 4.8 %)	14 (0.09 M, 0.2 %)	11 (0.06 M, 0.1 %)	<0.0001
No	8226 (92.5 M, 87.1 %)	1720 (13.1 M, 12.3 %)	53 (0.5 M, 0.5 %)	38 (0.1 M, 0.09 %)	
**Alcohol use**					
Men: 0–2 /Women: 0-1drink/day	1891 (29.3 M, 22.4 %)	386 (3.6 M, 24.1 %)	9 (0.08 M, 14.0 %)	6 (0.05 M, 21.4 %)	<0.0001
Men: >2 /Women: >1 drink/day	3481 (45.2 M, 34.6 %)	265 (2.3 M, 15.4 %)	16 (0.1 M, 22.3 %)	14 (0.05 M, 22.6 %)	
**MetS (%)**	2046 (21.0 M, 82.3 %)	525 (4.4 M, 17.1 %)	13 (0.1 M, 0.5 %)	10 (0.05 M, 0.2 %)	<0.0001
**DM Status (%)**					
No DM	7443 (97.3 M, 92.2 %)	868 (7.8 M, 7.4 %)	36 (0.2 M, 0.2 %)	29 (0.2 M, 0.2 %)	<0.0001
Pre-DM	2792 (26.0 M, 83.3 %)	726 (5.0 M, 16.1 %)	21 (0.2 M, 0.6 %)	5 (0.02 M, 0.1 %)	
DM	1014 (7.4 M, 74.8 %)	367 (2.3 M, 23.1 %)	10 (0.2 M, 1.7 %)	15 (0.05 M, 0.5 %)	
**NAFLD (%)**					
Yes	440 (4.7 M, 75.9 %)	132 (1.4 M, 22.9 %)	5 (0.06 M, 1.0 %)	5 (0.008 M, 0.1 %)	<0.0001
No	10,809 (126.0 M, 89.7 %)	1829 (13.7 M, 9.8 %)	62 (0.5 M, 0.4 %)	44 (0.2 M, 0.1 %)	
**Obese (%)**					
Yes	2887 (28.3 M, 88.0 %)	458 (3.7 M, 11.6 %)	9 (0.09 M, 0.3 %)	11 (0.04 M, 0.1 %)	<0.0001
No	8362 (102.4 M, 89.5 %)	1503 (11.4 M, 10.0 %)	58 (0.5 M, 0.4 %)	38 (0.2 M, 0.2 %)	

Data displayed as unweighted number, weighted number and weighted percentage for categorical variables, and weighted means ± SE for continuous variables.

p-value will be calculated by comparing values among FIB-4 groups.

Abbreviations: DM = Diabetes Mellitus; NAFLD=Nonalcoholic Fatty Liver Disease; BMI=Body Mass Index; LDL-C = Low density Lipoprotein Cholesterol; HDL-C = High Density Lipoprotein Cholesterol; SBP = Systolic Blood Pressure; DBP=Diastolic Blood Pressure; MetS = Metabolic Syndrome.

**Percentages are recorded as row percentages.

Table 3 showed results from multiple logistic regression analyses. Overall, older adults were more likely to have a high FIB-4 score (odds ratio [OR] per 10 years =1.08, 95 % CI 1.05–1.12, *p* < 0.0001), with females having a 57 % higher likelihood of having high FIB-4 (OR = 1.57, 95 % CI 1.00–2.47, *p* < 0.05). Specifically, non-DM persons with higher total cholesterol were less likely to have high FIB-4 (OR = 0.49, 95 % CI 0.32–0.75, *p* < 0.01) whereas those with NAFLD were more likely to have high FIB-4 relative to those without NAFLD (OR = 8.36, 95 % CI 1.77–39.53, p < 0.01). Non-Hispanic black persons with DM had a 90 % lower likelihood of having a high FIB-4 score compared to non-Hispanic whites (OR = 0.10, 95 % CI 0.01–0.66, *p* < 0.05).

**Table 3:** Multiple logistic regression examining risk factors and the odds of high or very high FIB-4 level in those with and without DM, NHANES III.

	Odd ratio (95 % confidence interval)		
	Total	Non-DM	DM
Age (per 10 years)	1.08 (1.05–1.12)⁎⁎⁎	1.11 (1.09–1.12)⁎⁎⁎	1.02 (0.95–1.10)
Gender (female vs. male)	1.57 (1.00–2.47)⁎	1.41 (0.84–2.35)	3.23 (1.13–9.30)⁎
Ethnicity			
Non-Hispanic White (ref)	Reference	Reference	Reference
Mexican American	0.87 (0.45–1.71)	1.12 (0.49–2.61)	0.37 (0.0.9–1.60)
Non-Hispanic Black	0.95 (0.47–1.93)	1.54 (0.80–2.94)	0.10 (0.01–0.66)⁎
Other	0.77 (0.13–4.54)	1.12 (0.16–7.78)	0.20 (0.02–2.44)
Total cholesterol (per SD)	0.69 (0.44–1.06)	0.49 (0.32–0.75)⁎⁎	1.09 (0.69–1.71)
Current smoker (yes vs. no)	1.17 (0.56–2.44)	1.70 (0.86–3.34)	0.22 (0.04–1.12)
Alcohol use			
Mild vs. None	0.93 (0.45–1.95)	1.04 (0.47–2.30)	0.68 (0.15–3.19)
Heavy vs. None	1.71 (0.70–4.18)	1.68 (0.74–3.84)	2.07 (0.18–24.26)
Metabolic syndrome			
Neither MetS nor DM	Reference	N/A	N/A
MetS without DM	0.84 (0.42–1.69)	N/A	N/A
With DM	2.38 (0.83–6.87)	N/A	N/A
NAFLD (yes vs. no)	2.44 (0.71–8.37)	8.36 (1.77–39.53)⁎⁎	0.46 (0.07–3.19)

⁎ p < 0.05.

⁎⁎ p < 0.01.

⁎⁎⁎ p < 0.0001.

**Fig. 1:**
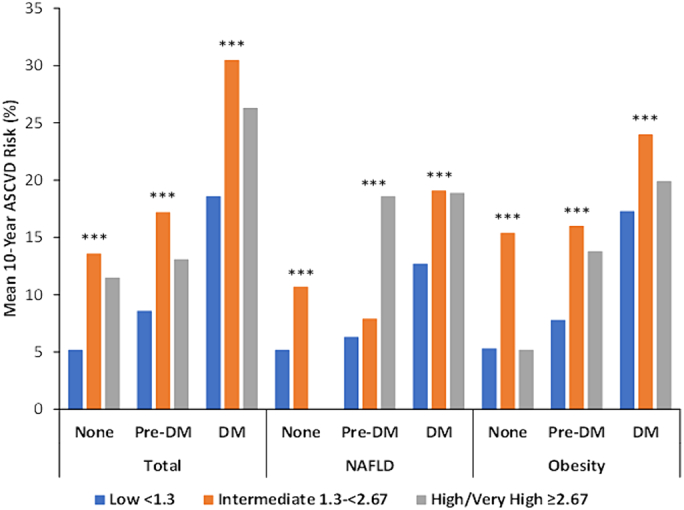
Mean 10-year atherosclerotic cardiovascular disease risk by FIB-4 category among persons with and without pre-DM or DM for NHANES III. ^⁎⁎⁎^ p<0.0001.

**Fig. 2:**
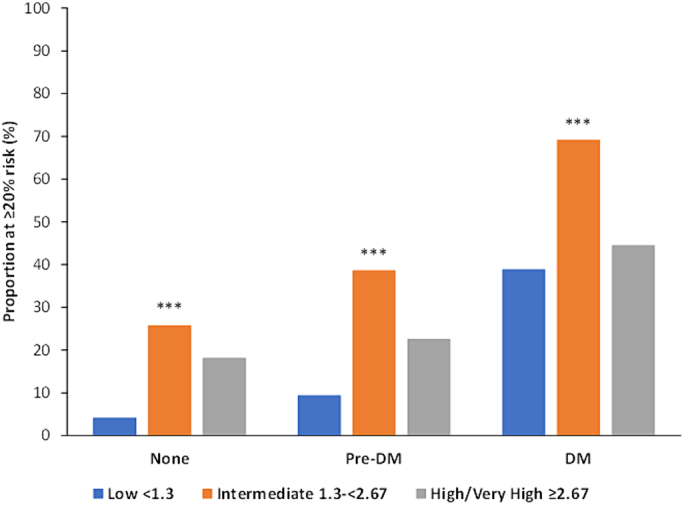
Proportion of adults at ≥20 % ASCVD risk among persons with and without pre-DM or DM according to FIB-4 category for NHANES III. ^⁎⁎⁎^ p<0.0001.

The mean 10-year ASCVD risks in the overall sample were 18.6 %, 30.5 %, and 26.3 % for DM patients; 8.6 %, 17.2 %, and 13.1 % for pre-DM patients; and 5.2 %, 13.6 %, and 11.5 % for non-DM patients for those with low, intermediate, and high or very high FIB-4 scores, respectively (*p* < 0.0001 across all diabetes categories). Those with NAFLD or obesity showed similar trends across diabetes and FIB-4 subgroups (all *p* < 0.0001) (Table 4) (Fig. 1). Those with low FIB-4 scores tended to be at lowest estimated risk of ASCVD, while those with intermediate FIB-4 scores were at greater risk (all *p* < 0.0001). The proportion of those with DM who had estimated 10-year ASCVD risks ≥20 % were 38.9 %, 69.2 %, and 44.5 % for those with low, intermediate, and high or very high FIB-4 scores, respectively (*p* < 0.0001). For those without DM only 4.2 %, 25.8 %, and 18.2 % were at ≥20 % ASCVD risk among those with low, intermediate, and high or very high FIB-4, respectively (p < 0.0001) (Fig. 2).

**Table 4:** 10-year risk of ASCVD according to low, intermediate, high/very high levels of FIB-4 in those with NAFLD or obesity, with and without pre-DM / DM, NHANES III.

Total (*n* = 6582; 71.7 M)					NAFLD (n = 348; 3.7 M)					Obesity (n = 1961; 18.9 M)				
FIB-4 (by risk groups)	None (*n* = 3261; 43.3 M)	Pre-DM (*n* = 2282; 21.0 M)	DM (*n* = 1039; 7.4 M)	Total (n = 6582; 71.7 M)	FIB-4 (by risk groups)	None (*n* = 86; 1.3 M)	Pre-DM (n = 131; 1.3 M)	DM (*n* = 131; 1.1 M)	Total (*n* = 348; 3.7 M)	FIB-4 (by risk groups)	None (*n* = 726; 8.6 M)	Pre-DM (*n* = 753; 6.6 M)	DM (*n* = 482; 3.7 M)	Total (*n* = 1961; 18.9 M)
Low (*n* = 5208; 59.0 M)	5.2 ± 0.2	8.6 ± 0.3	18.6 ± 0.9	7.4 ± 0.2	Low (*n* = 227; 2.4 M)	5.2 ± 0.4	6.3 ± 0.3	2.7 ± 1.0	7.8 ± 0.5	Low (*n* = 1578; 15.6 M)	5.3 ± 0.3	7.8 ± 0.4	17.3 ± 1.0	8.4 ± 0.3
Intermediate (*n* = 1305; 12.2 M)	13.6 ± 0.6	17.2 ± 0.6	30.5 ± 1.7	17.3 ± 0.4	Intermediate (*n* = 114; 1.3 M)	0.7 ± 0.8	7.9 ± 0.9	9.1 ± 2.4	2.4 ± 1.1	Intermediate (*n* = 368; 3.3 M)	15.4 ± 1.0	16.0 ± 1.1	24.0 ± 2.5	17.7 ± 0.7
High and very high (*n* = 69; 0.5 M)	11.5 ± 1.9	13.1 ± 3.8	26.3 ± 6.8	15.7 ± 2.4	High and very high (n = 7; 0.06 M)	N/A	8.6 ± 7.2	8.9 ± 2.7	8.7 ± 7.0	High and very high (n = 4; 0.02 M)	5.2 ± 0.9	13.8 ± 0.2	19.9 ± 7.7	15.9 ± 4.6
Total (n = 6582; 71.7 M)	6.5 ± 0.2	10.2 ± 0.3	21.7 ± 0.9	9.2 ± 0.2	Total (n = 348; 3.7 M)	7.4 ± 0.6	7.1 ± 0.3	5.1 ± 1.2	9.6 ± 0.6	Total (n = 1961; 18.9 M)	6.8 ± 0.3	9.3 ± 0.4	18.8 ± 1.0	10.0 ± 0.3
p-value	<0.0001	<0.0001	<0.0001	<0.0001	p-value	0.0001	0.3614	0.0120	0.0032	p-value	<0.0001	<0.0001	0.0639	<0.0001

**Fig. 3:**
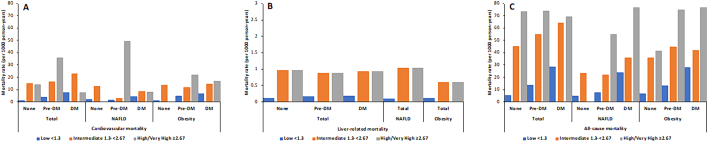
Mortality rates per 1000 person years for a) cardiovascular, b) liver-related, and c) all-cause mortality among US adults with and without Pre-DM or DM.

Fig. 3 showed CVD, liver-related, and all-cause mortality rates per 1000 person-years according to the presence or absence of pre-DM or DM across FIB-4 categories for the total sample as well as for those with NAFLD or obesity. In the total sample, those with high or very high levels of FIB-4 tended to have the highest CVD mortality rates (17.7) followed by those with intermediate FIB-4 (16.9) then low FIB-4 levels (2.0). While a similar trend was observed across all diabetes subgroups, non-DM and DM patients with intermediate FIB-4 scores had the highest CVD event rates (15.3 and 23.3, respectively). In persons with high FIB-4 scores, those with pre-diabetes had higher CVD event rates (36.1) than those with (8.0) or without (14.0) DM. There were similar findings in NAFLD and obesity patients, but for obese patients with diabetes, those with high FIB-4 scores (16.8) had the greatest CVD mortality rate compared to those with low (6.6) and intermediate (14.7) FIB-4 scores. Liver-related and all-cause mortality data showed more stable trends with lower FIB-4 scores and no diabetes having the lowest event rates and higher FIB-4 scores and diabetes having the highest event rates.

Table 5 showed the unadjusted and adjusted hazard ratios (HRs) and 95 % confidence intervals (CIs) for CVD and all-cause mortality. These results demonstrated non-DM adults with high FIB-4 scores tended to have the highest risk of CVD and all-cause mortality relative to pre-DM and DM adults with low FIB-4 scores (reference group) after adjusting for covariates. CVD mortality data showed those with high or very high versus low FIB-4 scores had unadjusted HRs of 14.01 (5.63–34.90), 8.76 (3.66–20.95), and 0.89 (0.22–3.53) in non-DM, pre-DM, and DM patients, respectively. Adjustment for covariates largely attenuated our findings. Further, those with high or very high versus low FIB-4 scores also showed increased HRs for total mortality in non-DM, pre-DM, and DM patients, respectively: 15.63 (9.36–26.11), 5.46 (3.16–9.43), and 2.07 (0.90–4.74), which were also attenuated after adjustment. However, in the overall sample the risk of total mortality remained significant after adjustment in those with high or very high versus low FIB-4 scores: HR = 1.66 (1.20–2.31).

**Table 5:** Hazard ratio of cardiovascular and all-cause mortality according to FIB-4 groups stratified by metabolic status, NHANES III.

	Total (n = 13,326; 146.7 M)				NAFLD (*n* = 640; 6.7 M)				Obesity (*n* = 3365; 32.2 M)			
**Cardiovascular mortality (unadjusted)**												
**FIB-4 (by risk groups)**	None	Pre-DM	DM	Total	None	Pre-DM	DM	Total	None	Pre-DM	DM	Total
Low	Ref	Ref	Ref	Ref	Ref	Ref	Ref	Ref	Ref	Ref	Ref	Ref
Intermediate	3.17‡ (9.32–18.62)	4.21‡ (3.02–5.87)	3.21‡ (2.21–4.65)	8.43‡ (6.86–10.34)	5.20† (1.75–15.49)	1.80 (0.34–9.64)	1.78 (0.73–4.32)	3.08† (1.37–6.94)	12.51‡ (5.66–27.65)	2.69† (1.51–4.79)	2.18† (1.32–3.62)	5.07‡ (3.59–7.15)
High / Very High	14.01‡ (5.63–34.90)	8.76‡ (3.66–20.95)	0.89 (0.22–3.53)	8.81‡ (4.61–16.84)	N/A	7.07† (3.48–210.3)	1.15 (0.11–12.40)	2.81† (2.39–68.66)	N/A	5.54 (0.45–68.01)	2.81 (0.51–15.48)	4.83⁎ (1.14–20.5)
**Cardiovascular mortality (adjusted)**												
**FIB-4 (by risk groups)**	None	Pre-DM	DM	Total	None	Pre-DM	DM	Total	None	Pre-DM	DM	Total
Low	Ref	Ref	Ref	Ref	Ref	Ref	Ref	Ref	Ref	Ref	Ref	Ref
Intermediate	1.00 (0.15–6.51)	0.80 (0.59–1.07)	1.24 (0.70–2.22)	1.05 (0.82–1.35)	0.31 (0.03–2.78)	0.85 (0.23–3.18)	0.46 (0.12–1.71)	0.55 (0.24–1.27)	2.42 (0.94–6.22)	0.69 (0.36–1.32)	0.90 (0.36–2.22)	1.03 (0.66–1.59)
High / Very High	N/A	1.71 (0.84–3.46)	0.58 (0.15–2.19)	1.34 (0.74–2.42)	0.03 (0.00–15.98)	4.11 (0.75–22.34)	0.45 (0.12–1.67)	1.55 (0.47–5.15)	N/A	2.45 (0.18–33.84)	0.78 (0.18–3.44)	1.35 (0.42–4.37)
**All-cause mortality (unadjusted)**												
**FIB-4 (by risk groups)**	None	Pre-DM	DM	Total	None	Pre-DM	DM	Total	None	Pre-DM	DM	Total
Low	Ref	Ref	Ref	Ref	Ref	Ref	Ref	Ref	Ref	Ref	Ref	Ref
Intermediate	8.30‡ (6.90–9.97)	4.16‡ (3.51–4.93)	2.41‡ (1.94–3.00)	6.42‡ (5.67–7.27)	4.61† (1.76–12.11)	3.15⁎ (1.33–7.48)	1.38 (0.58–3.30)	2.76‡ (1.75–4.36)	5.59‡ (3.89–8.03)	3.85‡ (2.92–5.06)	1.51 (0.98–2.31)	4.01‡ (3.23–4.99)
High / Very High	15.63‡ (9.36–26.11)	5.46‡ (3.16–9.43)	2.07 (0.90–4.74)	9.12‡ (6.22–13.38)	N/A	8.30⁎ (1.03–66.72)	2.04† (1.26–3.29)	5.87† (1.78–19.33)	6.77⁎ (1.17–39.30)	6.90† (1.71–27.90)	2.85‡ (1.99–4.09)	6.53‡ (4.38–9.75)
**All-cause mortality (adjusted)**												
**FIB-4 (by risk groups)**	None	Pre-DM	DM	Total	None	Pre-DM	DM	Total	None	Pre-DM	DM	Total
Low	Ref	Ref	Ref	Ref	Ref	Ref	Ref	Ref	Ref	Ref	Ref	Ref
Intermediate	1.07 (0.87–1.30)	1.04 (0.90–1.20)	0.93 (0.70–1.24)	1.03 (0.91–1.17)	0.40 (0.09–1.73)	3.10 (0.81–11.77)	0.27† (0.11–0.65)	0.97 (0.64–1.47)	1.04 (0.70–1.52)	1.23 (0.80–1.87)	0.69 (0.39–1.22)	0.98 (0.74–1.29)
High / Very High	1.91⁎ (1.07–3.41)	1.36 (0.93–1.98)	1.26 (0.63–2.53)	1.66† (1.20–2.31)	N/A	5.56⁎ (1.01–30.75)	0.65 (0.28–1.54)	0.91 (0.34–2.47)	2.31 (0.42–12.71)	3.26 (0.95–11.18)	1.14 (0.70–1.86)	2.10⁎ (1.19–3.71)

Adjusting for age, sex, ethnicity, BMI, non-HDL-C, HDL—C, systolic and diastolic BP, cigarette smoking, and alcohol use.

⁎ p < 0.05.

† p < 0.01.

‡ p < 0.0001.

Similarly, we found that those without DM with intermediate, high, or very high FIB-4 scores were more prone to a higher risk of liver-related mortality relative to those with pre-DM and DM. Compared to those with low FIB-4 scores, those with intermediate, high, or very high FIB-4 scores had an increased risk of liver-related mortality overall, as well as in those with NAFLD or obesity. These findings were slightly attenuated after adjusting for covariates. In the overall sample, unadjusted HRs of 8.5 (95 % CI, 3.1–23.3; *p* < 0.0001), 6.6 (95 % CI, 1.5–30.4; *p* < 0.05), and 4.6 (95 % CI, 1.2–18.3; p < 0.05) were found for liver-related mortality comparing intermediate, high, or very high FIB-4 scores versus low FIB-4 scores for non-DM, pre-DM, and DM patients, respectively. Unadjusted HRs for liver-related mortality were 10.2 (95 % CI, 3.1–33.5; *p* < 0.01) for those with NAFLD and 7.1 (95 % CI, 1.5–32.3; *p* < 0.05) for those with obesity. In our adjusted analyses, these trends persisted with HRs of 6.8 (95 % CI, 1.4–32.9; p < 0.05), 4.6 (95 % CI, 0.6–39.1), and 3.6 (95 % CI 1.3–10.1; p < 0.05) comparing intermediate, high, or very high FIB-4 versus low FIB-4 scores in the total sample for non-DM, pre-DM, and DM patients, respectively. Adjusted HRs for liver-related mortality were 12.2 (95 % CI, 2.1–72.1; *p* < 0.01) for those with NAFLD and 4.1 (95 % CI, 0.6–29.0) for those with obesity.

## 4 Discussion

Our study is unique in examining the U.S. estimated prevalence of liver fibrosis in people with and without pre-DM or DM as well as in those with NAFLD or obesity. We find that advanced liver fibrosis is more prevalent in those with versus without DM. The presence of DM with higher FIB-4 scores is associated with especially high estimated ASCVD risks. Older age is significantly associated with a greater likelihood of having a high level of FIB-4. However, females and non-Hispanic whites, compared to males and non-Hispanic blacks, respectively, are at greater likelihood of having high FIB-4. When analyzing mortality rates across DM categories, high or very high FIB-4 scores are associated with greater CVD, liver-related, and total mortality, with pre-DM and DM found to further increase these event rates.

In our study, we show the varying prevalence of advanced fibrosis across diabetes subgroups for the total sample along with NAFLD and obesity groups. While other studies have examined the prevalence of advanced fibrosis in those with DM, they were much more limited in sample size and did not examine mortality outcomes [[14], [15], [16]]. A more recent study by Park et al. [17] found DM but not pre-DM was associated with advanced liver fibrosis in the general South Korean adult population with proportions of significant fibrosis (indicated by a liver stiffness measurement ≥2.97 kPa) in no glucose intolerance, prediabetes, and diabetes groups were 3.1 %, 4.4 %, and 16.7 %, respectively (*p* < 0.001). Those with DM had a 3-fold adjusted odds of significant fibrosis that was not significantly greater in those with pre-DM. While we show more moderate elevations in FIB-4 to be more common in those with pre-DM, those with DM, in particular have greater elevations in FIB-4 and associated ASCVD and mortality risks, compared to those without these conditions. Others have found higher FIB-4 scores to be a predictor for a greater risk of decreased insulin secretion and thus a greater risk of developing pre-DM and subsequently DM in a non-diabetic sample of adults [18]. Moreover, advanced fibrosis assessed by elastrography has been reported to be present in 7 % to 29 % of DM patients without liver disease [19,20] There are more limited data on advanced fibrosis in those with pre-DM, with some showing higher levels of significant fibrosis in those with pre-DM [21] while others showing similar prevalence (13 %) compared to those with no glucose intolerance (12 %) [22].

Our stratification of mean 10-year ASCVD risk scores by FIB-4 scores and diabetes status is also a unique attribute of our study. The mean 10-year ASCVD risk is highest in those with pre-DM. The 2013 ACC/AHA guideline for calculating a 10-year ASCVD risk score has been used extensively in various studies [[23], [24], [25]] to assess CVD risk. Chun et al. used this guideline and underscored the relationship between advanced liver fibrosis and higher ASCVD risk along with the risk score's efficacy in predicting the risk of CVD history [23]. They also found significantly greater ASCVD risk in those with advanced fibrosis compared to those without. In another study evaluating ASCVD scores, the severity of liver fibrosis was an independent risk factor for higher ASCVD risk after adjusting for obesity. While these findings support the overall trend of greater ASCVD risk across FIB-4 categories, they do not depict the relationship between diabetes status and ASCVD risk.

CVD, liver-related, and all-cause event rates are significantly higher in those with DM with high or very high FIB-4 scores compared to those with or without pre-DM and with low FIB-4. A previous study has already shown higher liver fibrosis scores to be associated with increased CVD, liver-related, and all-cause mortality in NHANES III [3]. However, this study focused on the overall NHANES III sample and did not assess mortality among diabetes categories or according to NAFLD or obesity. A recent study examining the NHANES 2015–2016 cohort showed while NAFLD measured by fatty liver index was substantially more common in those who were overweight and obese, further augmented by the presence of diabetes, liver fibrosis based on FIB-4 levels >1.46 and > 2.67 was actually higher in non-obese compared to obese individuals, with or without diabetes [26]. This study, however, did not examine outcomes (e.g., cardiovascular, liver, and total mortality) associated with liver fibrosis in those with pre-DM or DM according to the presence of NAFLD or obesity. Tada et al. revealed a significant association between the progression of liver fibrosis severity and CVD mortality in those with NAFLD from a general hospital sample, but there was no analysis of mortality in those with obesity exclusively or across diabetes categories in general [27]. We showed non-diabetes patients with high FIB-4 scores were at the greatest risk of CVD and all-cause mortality in NHANES III, and similar results were seen for liver-related mortality. While NAFLD and obesity patients also had greater event rates across diabetes and FIB-4 subgroups, NAFLD patients with intermediate FIB-4 were at greater risk of CVD mortality than those with high FIB-4 when compared to those with low FIB-4.

The strengths of our study include NHANES being a U.S. population-representative sample of U.S. adults with standardized measurement of FIB-4 levels and CV risk factors. NHANES allows an association with the U.S. population via a weighting procedure to determine the estimated number of people with various risk factors, diseases, and FIB-4 scores. Considering the diversity among adults in NHANES III, we are able to study data for many ethnicities including non-Hispanic white, non-Hispanic black, Mexican American, and others. Although many self-reported measures such as prior history of CVD, for example, are largely validated by analysis of medical records and laboratory tests, there may be problems with patients self-reporting accurately. In addition, as NHANES is a population-based survey, we do not have corresponding measures of ultrasound or magnetic resonance elastography in our cohort. However, the diagnostic accuracy of FIB-4 measures has been demonstrated by others in relation to advanced fibrosis from biopsy determined NALFD [28], ultrasound [29] and magnetic resonance [30] elastography. In a more recent study of 1068 patients examining the role of FIB-4 in assessing hepatic fibrosis. C-statistics ranging from 0.78 to 0.82 for diagnosing advanced fibrosis are found with a cutpoint of 2.68 showing sensitivities and specificities for predicting advanced fibrosis of 70.7 % and 79.1 % and demonstrating 81 % of unnecessary work-ups can be avoided [31]. Finally, given both FIB-4 and calculated ASCVD risk include age in their derivation, age is an important factor explaining the association between these two measures.

In summary, we show a greater prevalence of increased FIB-4 levels in those with diabetes compared to those with pre-DM or no DM. Higher FIB-4 scores are also associated with an increased risk of CVD and total mortality in those with pre-DM, DM, or neither condition. These findings warrant the need for increased efforts to identify those with pre-DM or DM who may have or be at increased risk of liver fibrosis and associated mortality.

## Funding

This study was funded by a contract from Gilead Sciences to the University of California, Irvine.

## Ethical statement

Given this study used deidentified information from the NHANES database, this study was exempt from Institutional Review Board review. The institutional review board of the National Center of Health Statistics (NCHS), a division of the Centers for Disease Control and Prevention, approved the protocols for NHANES.

## CRediT authorship contribution statement

**Matthew Bang:** Writing – review & editing, Writing – original draft, Software, Formal analysis. **Wenjun Fan:** Writing – review & editing, Supervision, Data curation. **Nathan D. Wong:** Writing – review & editing, Supervision, Project administration, Methodology, Funding acquisition.

## Declaration of competing interest

The authors declare that they have no known competing financial interests or personal relationships that could have appeared to influence the work reported in this paper.
